# Beneficial Effects of Astragaloside IV for Hair Loss via Inhibition of Fas/Fas L-Mediated Apoptotic Signaling

**DOI:** 10.1371/journal.pone.0092984

**Published:** 2014-03-27

**Authors:** Mi Hye Kim, Sung-Hoon Kim, Woong Mo Yang

**Affiliations:** 1 College of Korean Medicine and Institute of Korean Medicine, Kyung Hee University, Seoul, Korea; 2 Cancer Preventive Material Development Research Center, College of Korean Medicine, Kyung Hee University, Seoul, Korea; Duke University Medical Center, United States of America

## Abstract

Apoptosis with premature termination of hair follicle growth induces several types of hair loss and is one of the crucial factors of hair loss. Astragaloside IV, which is a major component of *Astragalus membranaceus*, is a cycloartane triterpene saponin. Although an anti-apoptotic effect of Astragaloside IV has been reported, its effects against hair loss have not been investigated. To explore the underlying mechanisms of Astragaloside IV on apoptotic signaling in hair follicle, the dorsal skin of depilated C57BL/6 mice was topically treated with 1 and 100 μM Astragaloside IV for 14 days. In Astragaloside IV-treated group, TUNEL-positive cells were reduced. We found that Astragaloside IV blocked the procaspase-8, resulting in the inhibition of caspase-3 and procaspase-9 activities. The changes were accompanied with down-regulation of Bax and p53, and up-regulation of Bcl-2 and Bcl-_xL_ by Astragaloside IV treatment. In addition, activation of NF-κB and phosphorylation of IκB-α were inhibited, along with decreases in three MAPKs: ERK, SAPK/JNK and p38 by Astragaloside IV. The expressions of KGF, p21, TNF-α and IL-1β, which are keratinocyte terminal differentiation markers associated with catagen, were modulated by treatment with Astragaloside IV. These results demonstrated that Astragaloside IV is concerned with blocking the Fas/Fas L-mediated apoptotic pathway, which would be an alternative therapy for hair loss.

## Introduction

Hair follicle regression during catagen is characterized by apoptosis and terminal differentiation of the proximal epithelial hair bulb, perifollicular proteolysis, and matrix remodeling [1]. Disturbances in hair follicle cycling are represented by induction of apoptosis [2]. Apoptosis mediated by Fas/Fas ligand (Fas L) is accompanied with caspase activation and cytochrome c (Cyt c) release from mitochondria. B cell leukemia protein (Bcl)-2 family is involved in these signaling by controlling the intrinsic pathway of apoptotic pathway [3].

To date, two anti-hair loss drugs, finasteride and minoxidil, have been approved by the FDA [4]. However, new alternative and complementary treatments are required due to their undesirable side effects, low cure rate, and high recurrence rate.

Astragaloside IV is a main component of *Astragalus membranaceus*, a commonly used medicinal plant in East Asia [5]. It is a cycloartane triterpene saponin (C_41_H_68_O_14_, molecular weight = 784). Astragaloside IV is known to have anti-inflammation, anti-oxidant, anti-viral, anti-hyperglycemic, neuroprotection, gastroprotection, anti-chromic asthma and anti-apoptotic properties [6]. Astragaloside IV reduced apoptosis via regulation of p38 mitogen-activated protein kinase (MAPK) phosphorylation and caspase-3 activity, accompanied by a decrease in Bcl-2-associated X protein (Bax) expression and an increase in Bcl-2 expression in acute kidney injury rodent models [7]. In another study, Astragaloside IV prevented glucose-induced podocyte apoptosis partly through restoration of the balance of Bax and Bcl-2 expression and inhibition of caspase-3 activation [8]. Astragaloside IV also significantly prevented 1-methyl-4-phenylpyridinium ion-induced SH-SY5Y cell death via the inhibition of Bax-mediated pathways [9]. Although Astragaloside IV was reported to have anti-apoptotic activities under various pathophysiological conditions *in vivo* and *in vitro*, its effects on hair loss have not been clarified.

In this study, we used C57BL/6 mice with natural hair loss in the telogen phase determined by the pink color of the dorsum. Although chemotherapy-induced animal model has been widely used in the experiments related to hair loss, we suppose that naturally depilated mice are closer to human nature alopecia. The present study was designed to investigate the beneficial effects of Astragaloside IV on hair loss via inhibition of apoptosis signaling in mice.

## Materials and Methods

### Animal treatment protocol

Seven-week-old female C57BL/6 mice (18–20 g), with natural hair loss in the telogen phase (pink color), and normal mice were purchased from Samtako Experimental Animal Center (Ossan, Gyeonggi-do, Korea). Animals were divided into four groups: Normal, normal group without depilation; Vehicle, control group with depilation; A IV (1 μM), 1 μM of Astragaloside IV-treated group; and A IV (100 μM), 100 μM of Astragaloside IV-treated group (n = 7, respectively). Animals were given an unlimited amount of water and food and kept at a constant temperature (23°C) and humidity (55%) with a 12-h light/dark cycle during the experiment. All experiments were conducted according to the guidelines of the Guide for the Care and Use of Laboratory Animals of the National Institutes of Health. The protocol was approved by Committee on Care and Use of Laboratory Animals of the Kyung Hee University (Permit Number: KHUASP(SE)-13-046).

One-hundred microliters of 1 and 100 μM Astragaloside IV (PubChem CID: 13943297; Sigma, St. Louis, MO, USA) were applied topically on the upper back with depilation. Vehicle mice were topically applied 100 μL of vehicle (100% pure methanol) on the depilated skin. Normal mice did not receive any treatment. These procedures were performed once per day for 2 weeks. Following challenge for 15 days, photographs were taken and mice were sacrificed. Dorsal skins from mice were fixed in 4% formaldehyde or collected and frozen until use.

### Histology

Dorsal skin were fixed in formaldehyde for 24 h and transferred to phosphate-buffered saline (PBS). Fixed skins were hydrated with ethanol and PBS. Paraffin-embedded sections were cut every 4 μm. Hematoxylin and eosin (H&E) were applied to the section to monitor hair regrowth. Digital images were obtained using the Leica Application Suite (LAS) Microscope Software (Leica Microsystems Inc., IL, USA). The magnifications used were ×100, ×200 and ×400.

### Immunohistochemistry

Sections were boiled in 10 mM sodium citrate buffer for antigen unmasking. Slides were treated with 3% H_2_O_2_ for 30 min to reduce endogenous peroxidase and with normal goat serum to minimize non-specific binding. To detect apoptotic cells, commercially available terminal deoxynucleotidyl transferase dUTP nick end labeling (TUNEL) kit (Millipore, Billerica, MA) was used. After incubation with biotin-dUTP and terminal deoxynucleotidyl transferase (TdT), TUNEL-positive cells were visualized by anti-digoxigenin conjugate. To detect caspase-3-positive cells, the slide was treated by anti-mouse caspase-3 IgG (BD Pharmingen, San Jose, CA, USA) overnight at 4°C, and then biotinylated anti-rabbit antibody (BD Pharmingen) was applied for 1 h. Sections were then stained using the avidin/biotinylated enzyme complex (ABC) kit (Vector Laboratories, Burlingame, CA) and developed using the 3,3-diaminobenzidine (DAB; Dako, Glostrup, Denmark) substrate. Digital images were obtained using Leica Application Suite (LAS) Microscope Software (Leica Microsystems Inc., Buffalo Grove, IL, USA). The magnification used was ×100.

### Protein fraction

Whole-tissue protein fractions from skin tissues (100 mg, n = 7 per group) were obtained by homogenization in 2 mL T-PER tissue protein extraction reagent (Pierce, Rockford, IL, USA) containing a protease inhibitor cocktail (Roche, Indianapolis, IN, USA) on ice. Nuclear and cytoplasmic protein fractions from skin tissues (100 mg, n = 7 per group) were obtained by homogenization in cytoplasmic buffer (10 mM HEPES [pH 7.9], 10 mM KCl, 0.1 mM EDTA, 0.1 mM EGTA, 1 mM DTT, 0.15% Nonidet P-40, 50 mM β-glycerophosphate, 10 mM NaF, 5 mM Na3VO4), and the pellets were suspended in nuclear buffer (20 mM HEPES [pH 7.9], 400 mM NaCl, 1 mM EDTA, 1 mM EGTA, 1 mM DTT, 0.50% Nonidet P-40, 50 mM β-glycerophosphate, 10 mM NaF, 5 mM Na3VO4) containing protease inhibitor cocktail on ice for 15 min. The resulting homogenate was centrifuged at 12,000×g for 30 min at 4°C and supernatants were labeled whole-tissue, cytoplasmic or nuclear protein.

### Western blotting

The activation of β-actin, p21waf, keratinocyte growth factor (KGF), p53, extracellular signal-regulated kinase (ERK), phospho (p)-ERK, stress-activated protein kinase (SAPK)/c-Jun N-terminal kinase (SAPK/JNK), p-SAPK/JNK, p38, p-p38, caspases-3, -8, -9, p53, Bcl-2, Bcl-xL and Bax was investigated using the whole-tissue protein fraction. The nuclear protein fraction was used for assessment of the activation of nuclear factor (NF)-κB and the cytoplasmic protein fraction was used to determine IκB-α and p-IκB-α levels. Thirty micrograms of protein were electrophoresed in a 10% SDS-PAGE gel and transferred onto a PVDF membrane (Bio-Rad, Hercules, CA, USA). Each primary antibody (diluted 1∶1000 in 3% bovine serum albumin (BSA); Cell Signaling, USA) was then added to the membrane. Anti-rabbit alkaline phosphatase-conjugated secondary antibody (diluted 1∶2000 in 3% BSA; Santa Cruz, CA, USA) was used to detect the target proteins. After incubation for 2 h at room temperature, bound antibodies were visualized using an enhanced chemiluminescence (ECL) detection reagent (Amersham Pharmacia, Piscataway, NJ, USA). Relative band densities were determined using a computerized densitometry system.

### Enzyme-linked immunosorbent assay (ELISA)

Protein assay reagent (Bio-Rad, Hercules, CA, USA) was used to quantify protein concentrations under identical conditions. Tissue cytokine concentrations were quantified using a mouse tumor necrosis factor (TNF)-α, interleukin (IL)-1β ELISA kit (BD Bioscience, San Jose, CA, USA) according to the manufacturer's instructions. Cytokine levels were calculated as pg per mg total protein. The optical density was read at 450 nm using an enzyme-linked immunosorbent assay (ELISA) reader (Molecular Devices, Downingtown, PA).

### Statistical analysis

Significance was determined by one-way analysis of variance (ANOVA) and nonparametric tests. In all analyses, *P*<0.05 was taken to indicate statistical significance.

## Results

### Recovery of hair loss

**Figure 1:**
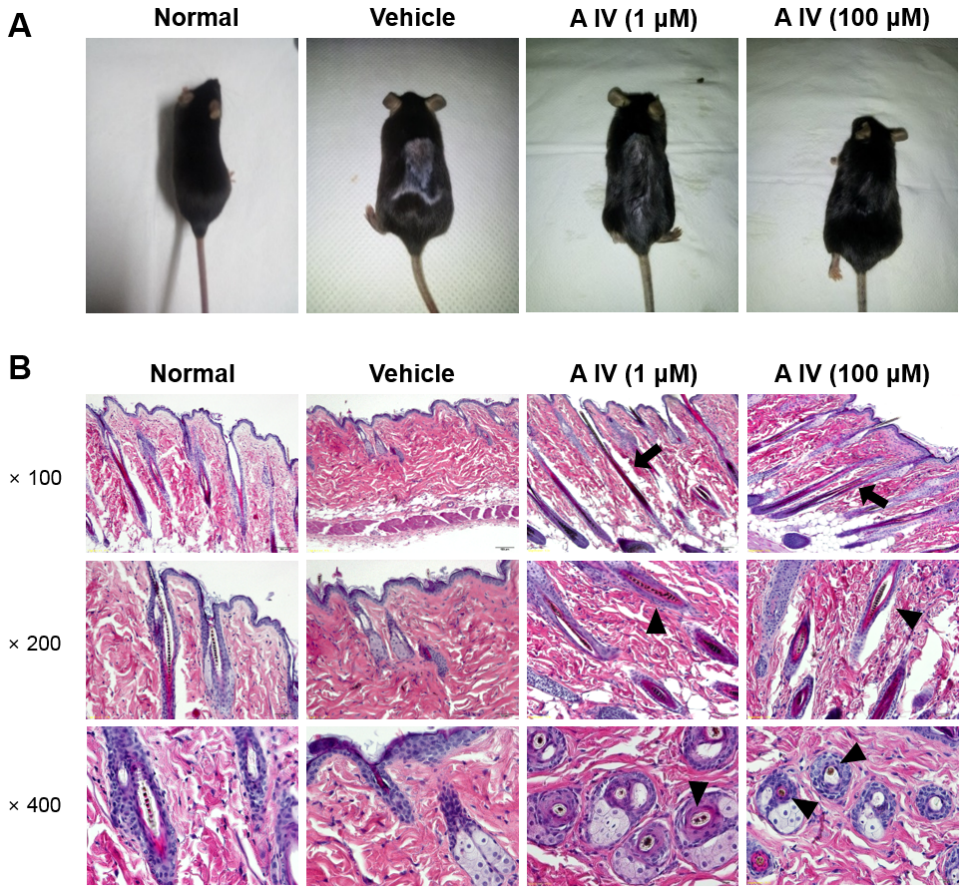
Morphological findings on the back of mice (A). Histological findings by hematoxylin and eosin (H&E) staining of dorsal skin sections (B). The magnification is ×100, ×200 and ×400. After treatment with Astragaloside IV, new hair shaft (arrows) and hair follicles containing hair fibers (arrowheads) appeared.

Topical application of Astragaloside IV resulted in near-complete recovery to normal levels in all mice, while the back of depilated mice retained a gray color in spontaneous catagen conditions (fig.1A). In addition, cutaneous color changes, indicating the inhibition of catagen and entry into anagen, occurred with Astragaloside IV.

In histology, spontaneous catagen with depilation induced dystrophic follicles along with fragmented hair shafts. Broken hair shafts did not emerge from hair follicles on the skin surface. Miniaturized hair follicles contained no hair fibers. Treatment with Astragaloside IV led to a recovery of tapering hair shaft length and broken hair follicles. New hairs on the surface of the epidermis appeared to be straight after Astragaloside IV treatment. Reversed miniaturized hair follicles showed hair fibers (fig.1B).

### Inhibition of apoptosis in hair follicles

**Figure 2:**
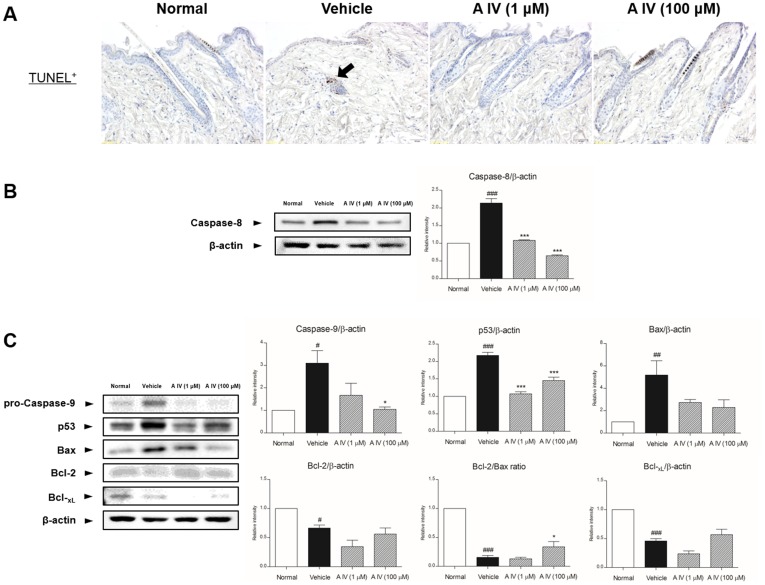
Expression of TUNEL-positive cells (A). Activation of caspase-8 (B), pro-caspase-9, p53, Bax, Bcl-2 and Bcl-xL (C). The magnification is ×200. Arrows point to TUNEL-positive cells. Results are presented as mean ±S.D. # and ## indicates the mean differs significantly between normal group and depilated group (p<0.05 and p<0.01, respectively). * indicates that the mean differs significantly between Astragaloside IV-treated group and depilated group (p<0.05).

**Figure 3:**
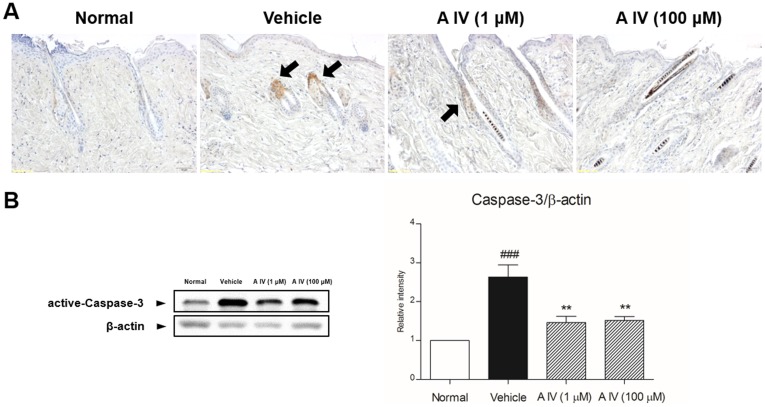
Expression of caspase-3-positive cells by immunohistochemistry (A) and activation of caspase-3 by western blot (B). The magnification is ×200. Arrows point to caspase-3-positive cells. Results are presented as mean ±S.D. ## indicates the mean differs significantly between normal group and depilated group (p<0.01). * indicates that the mean differs significantly between Astragaloside IV-treated group and depilated group (p<0.05).

The cell death pattern of hair follicles in catagen was indicated by TUNEL-positive cells with miniaturized follicles in the region of hair loss. In depilated mice, TUNEL-positive cells were seen in the epithelial cells surrounding the dermal papilla, but not in the dermal papilla of follicles (fig.2A). In Astragaloside IV-treated skin, few TUNEL-positive cells were seen in the hair follicles matrix as well as in dermal papilla (DP). Caspase-3-positive cells were also seen in hair follicles in the depilated region (fig.3A). Treatment with Astragaloside IV markedly decreased the number of caspase-3-positive cells with recovery of the hair shaft.

To explore the possible molecular mechanisms of inhibition of catagen by treatment with Astragaloside IV, apoptosis-associated protein expression was confirmed by Western blotting. The expression of caspases-3, -8 and -9 was decreased by 1 and 100 μM Astragaloside IV, but not in depilated mice, and there was a notable decrease in the level of Bax and p53 after treatment with Astragaloside IV in apoptotic catagen-affected mice (fig.2B, 2C and 3B).

The levels of Bcl-2, Bcl-xL and Bax expression were analyzed to evaluate effects on caspase activation. Astragaloside IV treatment reduced the expression of the proapoptotic factor Bax compared to control (fig.2C). Additionally, there were notable decreases in the levels of Bcl-2 and Bcl-xL. These results showed that the Bcl-2/Bax ratio was increased by Astragaloside IV, while it was decreased in catagen-affected skin.

**Figure 4:**
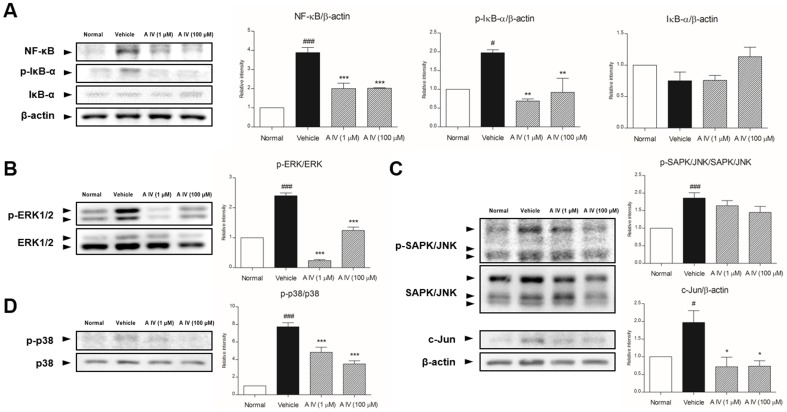
Activation of NF-κB and IκB-α (A), and three MAP kinase with c-Jun (B, C, D). Results are presented as mean ±S.D. # and ## indicates the mean differs significantly between normal group and depilated group (p<0.05 and p<0.01, respectively). * indicates that the mean differs significantly between Astragaloside IV-treated group and depilated group (p<0.05).

NF-κB and p-IκB-α expression levels in the skin of Astragaloside IV-treated mice were decreased, indicating that Astragaloside IV blocked the translocation of NF-κB to the nucleus and the phosphorylation of IκB-α (fig.4A). In addition, the levels of ERK, SAPK/JNK, and p38 were assessed in catagen-affected skin to investigate whether Astragaloside IV inhibits the phosphorylation of these three MAP kinases (fig.4B, 4C and 4D). Depilated skin contained high levels of ERK, SAPK/JNK and p38; these were decreased by Astragaloside IV. These decreases in MAP kinases were accompanied by dose-dependent reductions in the level of c-Jun.

### Modulation of terminal differentiation markers

**Figure 5:**
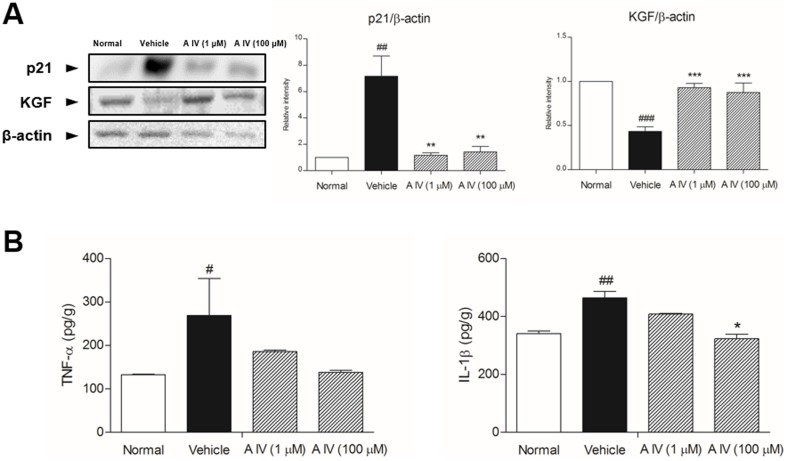
Expression of p21 and KGF (A), TNF-α and IL-1β. Results are presented as mean ±S.D. # and ## indicates the mean differs significantly between normal group and depilated group (p<0.05 and p<0.01, respectively). * indicates that the mean differs significantly between Astragaloside IV-treated group and depilated group (p<0.05).

Levels of keratinocyte premature differentiation markers, which indicate catagen, were evaluated in depilated skin. Figure 5A shows the protein expression of KGF, p21. Astragaloside IV induced KGF expression. Assessment of the level of p21 expression revealed decreases in treated mice.

In addition, we evaluated the protein levels of cytokines known to modulate hair growth and regression using whole-tissue lysates of dorsal skin tissue. Levels of TNF-α and IL-1β were increased in the depilated group (fig.5B). Astragaloside IV significantly reduced the level of IL-1β in a dose-dependent manner, compared to the depilated group. The level of TNF-α was decreased by Astragaloside IV treatment.

## Discussion

The premature termination of follicle growth and induction of apoptosis-regression in catagen leads to several types of hair loss [10]. Broken hair shafts, which are a result of abnormal hair cycles with induction of apoptosis, are not attached to hair follicles [1]. In this study, treatment with Astragaloside IV resulted in the emergence of new hair shafts from the skin surface and recoveries in the size of hair follicles and the length of hair shaft. Few TUNEL-positive cells were seen in hair follicle matrix as well as DP in Astragaloside IV-treated skin regions. These results demonstrate that Astragaloside IV inhibited apoptosis in hair keratinocytes, and retarded spontaneous catagen induction, resulting in the promotion of hair regrowth.

Caspase-dependent apoptotic cell death is triggered by the Fas/Fas L pathway [11]. Fas L triggers Fas receptor (Fas R), which transduces the signal by recruiting Fas associated death domain (FADD). Fas-FADD recruits procaspase-8 which combines to form the Fas-FADD procaspase-8 complex [12]. Self-cleavage of procaspase-8 activates caspase-8 as an initiator caspase of apoptotic cell death [13]. Caspase-8 induces release of Cyt c and concurrently activates caspase-3. Release of Cyt c into the cytoplasm recruits the apoptosis initiator enzyme procaspase-9, which promotes the formation of the active form of caspase-3 [3]. Thus, caspase-3 links the apoptotic pathways [14]. Astragaloside IV inhibited the activation of caspase-3 and procaspase-9 by blocking the cleavage of procaspase-8 suggesting that Astragaloside IV inhibited the Fas/Fas L-mediated apoptotic pathway through caspase-dependent signaling.

In addition, the intrinsic apoptotic pathway depends on the release of Cyt c through procaspase-8 [3]. Two types of Bcl-2 family members regulate the intrinsic death pathway, which induces apoptosis through interaction with pro-apoptotic Bax and anti-apoptotic Bcl-2 by regulating the release of Cyt c [15]. Bcl-2 and Bcl-_xL_ protect cells from apoptosis in the outer root sheath, resulting in promoting the hair follicle anagen-catagen transition, while Bax induces cell death [3]. Thus, the Bcl-2/Bax ratio in the hair matrix is markedly decreased during catagen [16]. Administration of Astragaloside IV onto depilated skin attenuated the expression of Bax, in contrast to Bcl-2. Bcl-2 and Bcl-_xL_ protein levels were increased by Astragaloside IV. These changes were accompanied by down-regulation of p53, which regulates the intrinsic apoptotic pathway in the hair matrix by promoting the expression of Bax during catagen [17]. Astragaloside IV inhibited p53 transcription along with Bax, and so regulated apoptosis through the mitochondrial intrinsic death pathway.

NF-κB plays an essential role in the control of apoptosis, as well as cell proliferation and differentiation [18]. Activation of NF-κB in response to several pro-apoptotic stimuli leads to phosphorylation and degradation of IκB-α in the cytoplasm. Concurrently, NF-κB translocates to the nucleus and targets gene transcription [19]. Astragaloside IV inhibited the translocation of NF-κB into the nucleus and the phosphorylation and degradation of IκB-α. In addition, levels of three MAPKs, which are involved in apoptosis, proliferation and inflammation in various skin diseases, were decreased by Astragaloside IV treatment [20].

Premature differentiation of keratinocytes, along with apoptosis, is a characteristic of catagen. The control of keratinocyte differentiation depends on a sudden decline of growth factors due to hair follicle transition from anagen to catagen. Hair fiber formation ceases due to a dramatic reduction in the proliferation of hair matrix keratinocytes. A previous report showed that KGF is secreted by fibroblasts, localized in the DP of hair follicles, and involved in follicular proliferation [1]. In our study, Astragaloside IV increased KGF protein levels. The activity of p21waf, which is associated with the control of keratinocyte growth and differentiation was inhibited by Astragaloside IV [21]. Additionally, TNF-α and IL-1β lead to the formation of dystrophic hair follicles and abnormal differentiation, resulting in potent inhibition of hair follicle growth [22]. Astragaloside IV also reduced the TNF-α and IL-1β protein levels.

**Figure 6:**
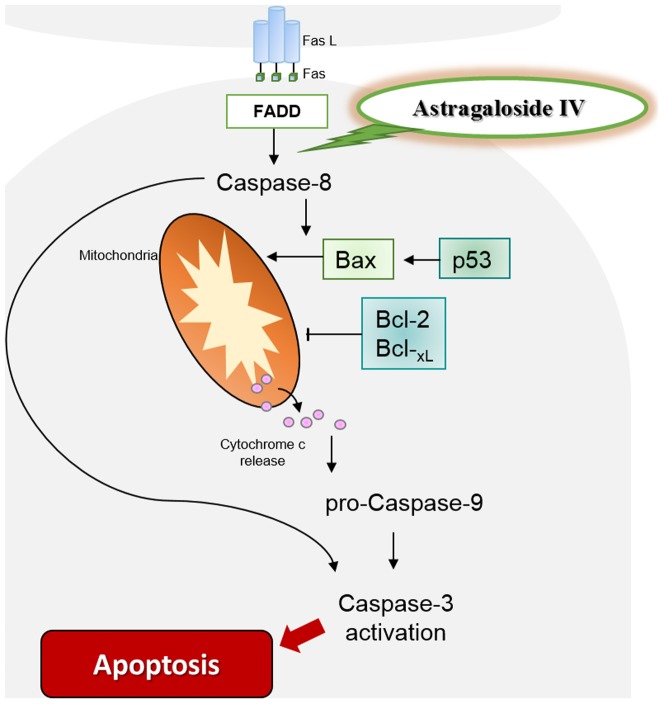
Diagram illustrates the potential actions of Astragaloside IV on Fas/Fas L apoptotic pathway. Astragaloside IV appear to block the cleavage of procaspase-8. As a result of the actions of Astragaloside IV, the release of Cyt c is inhibited, resulting in decreases of procaspase-9 and caspase-3. These activities lead to inhibition of apoptosis in hair keratinocyte. Fas/Fas L, Fas/Fas ligand; Cyt c, cytochrome c.

In summary, Astragaloside IV inhibited apoptosis-regression catagen in hair follicles via the Fas/Fas L-mediated cell death pathway (fig.6). Furthermore, terminal differentiation of hair keratinocytes, along with levels of growth factors and cytokines, were attenuated by Astragaloside IV, leading to hair regrowth. Astragaloside IV may be used for treatment of hair diseases such as alopecia, effluvium and hirsutism by retarding spontaneous catagen via an inhibition of caspase-dependent cell death triggered by the Fas/Fas L apoptotic pathway.
